# Discussions of the Mississippi Valley Dental Association

**Published:** 1876-08

**Authors:** F. W. Sage


### DISCUSSIONS OF THE MISSISSIPPI VALLEY DENTAL ASSOCIATION.

REPORTED BY F. W. SAGE, D. D. S.

### WEDNESDAY EVENING SESSION.

Dr. Taft, continued: A gentleman recently reported to me a case where he had extracted and replanted a tooth, the pulp of which, as seen through the cavity of decay, is now alive. Do not remember the reason for its having been extracted. This, and one or two other cases of the kind, go to corroborate Dr. Watt's statement that nerve tissue may be reunited.] I infer that this is often the case in replanted teeth, because if it were not there would be marked evidence of deterioration which we fail to discover. The nutritive process is evidently re-established. It has been offered, in explanation, that the new growth is fungous. This is an error, for the reproduced tissue is found to perform all the functions of the original nerve. Dr. Taft urged the importance of preserving *data* for our future guidance.

Dr. Morrison: I should have no hesitation in performing the operation of replanting a tooth for almost any person, regardless of systemic conditions, unless there was extreme debility. I should not fear to transplant teeth, as far as the danger of transmitting disease is concerned; if the periosteum be scraped off that danger is avoided.

Dr. Berry spoke of the care which physicians employ in obtaining pure vaccine matter. As to the danger of transmitting diseases (which it is proposed to avoid by scraping off the periosteum of the root,) is not *syphilis*, for instance, in the bones?

Dr. Cravens: In the winter of 1872, I extracted an upper and a lower molar and replanted them. Did not shorten the roots nor drill out the sockets. (If the teeth be replaced soon, this will be found to be unnecessary.) Both operations were successful; in two weeks the teeth were firm. I became emboldened by my success, so that subsequently finding it impossible, for some reason, to get into the canal of an incisor I was treating, for a lady forty-two years old, I extracted the tooth and inserted an artificial tooth which I had prepared for the occasion. It seemed to become firm in the socket, but the operation finally proved to be a failure.

Subject passed.

The first subject on the programme was next opened. "Dental Therapeutics."

Dr. Watt referred to a pale, anæmic condition of the gums in the case of young ladies. The treatment must be directed to an improvement of the general health.

Dr. Taft: There should be a more perfect knowledge of the action of remedies in common use. Dentists often betray a lamentable ignorance of the properties of the simplest remedies. Escharotics are used, diluted or in full strength, indiscriminately, whether it be to disinfect a pulp cavity or to cut down hypertrophied tissue. The dispensary should be studied for a knowledge of these agents.

We must learn the definite action of a remedy before we can use it intelligently. When elixir vitriol was first brought to our notice, there were very many, and are still, who did not understand its mode of action. Some asserted that it would produce death of the tissues; others, that it would act upon diseased parts but not affect a healthy surface. These diverse opinions came from men prominent in the profession. The simple fact is, elixir vitriol will dissolve, readily, any calcareous substance, Upon healthy living tissue it has but little effect, but upon degenerated tissue it acts with considerable energy.

Salicylic acid is a valuable antiseptic. Whether or not it is the best disinfectant may be a question. I have used it for a year and a half, and am much pleased with its operation.

Dr. Smith spoke of the treatment of sensitive dentine, and said: "I have almost entirely abandoned local treatment of sensitive dentine, because I have but little faith in it. We must look to constitutional treatment. Drs. Garretson and Flagg have discussed this matter fully. Assafetida, morphia and bromide of potassium have bcen recommended. The latter is the great remedy in nervous affections. Assafetida, in some cases where I have prescribed it, has had a very happy effect.

"One matter in particular demands our attention, and that is the treatment of girls during the catamenial period. Much has been said about the overtaxing of young girls at that period and the effect upon the nervous system. Dr. Clark has discussed the subject very thoroughly in his late publication, 'Building of a Brain.' I have noticed that during the cantamenial period our patients have less power of endurance than at other times. The operation should be postponed until a more favorable period.

"I think, if the patient is a school girl, that all dental operations should be performed (if possible to so arrange it) during her vacation. I would not resort to medication, but would advise the observance of the strictest hygienic laws. If there is a lack of tonicity, send the girl to the country. Bromide of potassium is prescribed by many for nervous diseases. It is a treacherous drug. Dr. Flagg says, 'I do oppose its general use. I am also opposed to the use of chloral, which some of my patients use habitually, and even bring it to the office with them. It is not only treacherous but dangerous.

Dr. Watt: This is the most important subject on the programme. It ought to be made the subject for a meeting, in which every other subject should be ruled out. As Dr. Taft has said in regard to the use of therapeutic agents, "we have been going it blind." I can remember when all therapeutic agents were used indiscriminately. At a meeting of this Association many years ago, the subject of the modus operandi of tannin, nitrate of silver, etc. was presented for consideration. Some of the older members appeared to shrink from a consideration of the subject, said that the dental profession was not able to grapple with it. It was then made a subject for consideration for the next meeting, with the request that any who were ready, to report through the "REGISTER," need not wait for the next meeting. Since that time there has been considerable progress made. There are some things, however, that remain to be learned.

Dr. Smith: I had a patient, an unmarried lady of adult years, who on two occasions (I had operated for her a dozen times or more) showed a peculiar irritability. She was spiteful and would have struck me had she not possessed too much good sense. Her father spoke about it, said she acted strangely at home sometimes. I asked, "had she dismenorrhœa?" He said he believed she had. I am convinced now that she had some uterine trouble.

I would recommend to every one to read Dr. Clark's "Sex and Education," also "The Building of a Brain," an essay read before the teachers at Detroit.

Dr. Baxter: The white decay I treat constitutionally, almost as I would treat a case of chills and fever, with the addition of the hypophosphates. I have treated persons whose teeth were soft and friable, and in the course of six to twelve months they became as hard as flint. Wheat is good food for such persons.

Dr. Watt: Dr. Flagg has said that the bromide of potassium is treacherous. A person is not so likely to form an appetite for it as for opium or tobacco, but if used persistently it fails to exert its benign effect. An occasional dose acts like a charm in reducing nervous irritability, In protracted operations I frequently prescribe it. In the ligation of hemorrohidal tumors I give it in large doses; the patient will not sleep but lies still and thinks. But suppose you find a patient who is affected with chronic sleeplessness, you will almost invariably find it impossible to operate on the teeth. Bromide of potassium if used habitually brings on a condition bordering on insanity; the patient becomes timid. I have seen a naturally brave woman reduced to a state of most abject timidity by the use of this drug. It will not do to trifle with it; it is very good for a few doses; it is not immediately so dangerous as chloral, nor in the same way. Dr, French, of Pittsburg, gives his patients a large dose of valerian for sensitiveness of dentine. After the lapse of five minutes he works with great rapidity. I have not resorted to that method of practice, but I was pleased with the results he achieved.

Dr. Corson related a case in which he had administered a dose of valerian for a lady, and had extracted her teeth. It operated like a charm in reducing nervous irritability.

Dr. Cravens: A girl twelve years old was affected with the following symptoms: sleeplessness; loss of appetite, and general debility; was under homœopathic treatment; her teeth were exquisitely sensitive and were affected with white decay. I filled one or two teeth and had to abandon the effort. I gave her doses of dry phosphate of lime for about two weeks, that being the only preparation of the kind I could get at the time. Afterwards I administered the syrup lactophosphate of lime three times daily. The girl's appetite improved, and in two or three weeks her father reported that she was much better. The sensitiveness of the teeth was very materially reduced, so that she bore the operation of filling very well indeed.

A lady thirty years old, whose teeth were affected by white decay, which extended under the gums in some cases, applied to me to have them filled. I could accomplish but little on account of the pain which I necessarily inflicted. She was pregnant, had passed the seventh month. After consulting her physician, and with his consent, I administered the lacto-phosphate lime, using about four ounces in two weeks. By that time the sensitiveness was very much reduced, and the nervous irritability very appreciably diminished. The superabundance of saliva, which I had at first noticed, had disappeared. She afterwards used the same preparation, administering it to her child, the development of whose teeth seemed to be retarded; it operated to hasten their eruption.

Dr. Taft: I apprehend that it was the lactic acid alone which diminished the sensitiveness.

Dr. Meredeth: A dentist applied to me for treatment of an ulcerated tooth which had been filled. Made a hypodermic injection of chloroform. The tissues puffed up in a remarkable manner; in a few days the parts began to slough, I feared the tooth and the process would be lost. However, healthy granulation at last set in.

FRIDAY MORNING SESSION.

Dr. McKellops, of St. Louis, occupied the morning in exhibiting some new appliances, including the diamond reamer, hard rubber discs, soft rubber discs with cloth linings to carry polishing powders, and a very hard and durable finishing stone. The hard rubber discs are invaluable for separating teeth, and are particularly useful in finishing fillings on approximal surfaces, leaving a remarkably smooth polished surface so that liitle or no further labor in polishing is required, The special merit of these discs is their durability, they seem altogether unaffected by months of use. One which the Dr. exhibited cut readily and rapidly through a block of porcelain teeth. They can be made according to the following directions:

English block rubber, one part.

Corundum (any No. from 50 to 100) three parts by weight.

Put these into a tea cup and set in a pan of boiling water, knead with two round ended sticks until all the corundum is incorporated in the rubber; mould into required shape and vulcanize about an hour.

These discs are not to be found at the depots. The credit of this receipt Dr. McKellops awards to his partner Dr. Bartleson.

A vote of thanks was offered Dr. McKellops for the interesting and instructive illustrations of the uses of these implements,

The fourth subject was next announced, "Filling Teeth. Comparative value of different methods of operating and of different materials."

Dr. Rawls spoke of the therapeutic influence of lead when used as a root filling. Its non-conducting properties also recommend it.

Dr. McKellops recommended the use of gutta percha dissolved in chloroform for filling difficult roots. It can be carried very perfectly to the apex of the root, and fills every crevice. He thinks it is by far the best root filling, excepting in cases, as in anterior teeth, where the root is easy of access and can be readily filled with gold. A broach dipped into the liquid is to be worked into the root and left until the chloroform partially evaporates, repeat this several times. Finally work in pieces of White's gutta percha, which will become somewhat softened by contact with the semi-fluid contents of the root and will act as a final stopping. Usually the operation can be performed in fifteen minutes, some patience is required.

Dr. Sage: One advantage of this preparation over any of the cements which have been used for root filling, is that it seems to be more easily induced to enter a minute orifice. A bit of floss silk saturated with it can be carried on a fine broach to the apex of a root into which it is often impracticable to force a string saturated with os-artificial, however thin. The latter preparation has a tendency to clog, while the liquid gutta percha slips easily into a tortuous root as though the surface were oiled.

Dr. Morrison spoke of the imperfection of root fillings of gold wire. Has had occasion to extract to cure abscess, and has made a careful examination of a good many such fillings. A better way is to carry up liquid oxychlo. zinc on the wire and pump it so as to fill every crevice, then force the wire up as far as possible. I have found after all my pains that the point of the wire does not in some cases reach the end of the root. In other cases it has protruded beyond the end. I would rather have this occur than to have it fall short of the apex. Much of the trouble from filled roots occurs because of a lack of care in cleansing them out thoroughly in the first place. I would not wait until an entire cure of abscess is effected, if there be a fistulous opening, before filling the roots.

Dr. McKellops: In many cases you can not force the wire into the root. If you use oxychlo.-zinc it is liable to set too quickly; but with a fine broach you can force the gutta percha preparation into any constricted opening. Try the experiment on an old tooth, with gold wire, os-artificial or any other material, and then try the solution of gutta percha, and note the difference in the results.

Dr. Watt: I find this preparation excellent for small roots but in large ones it shrinks too much.

Dr. McKellops: Use gold for your large roots, you don't want this preparation in such cases.

Dr. Dennis: I have been much pleased with the results I have obtained in using lead as a root filling. Have used tin, but have never been certain that I have carried it perfectly to the apex. A lead wire for filling a root is to be made of the proper size and shape, using a little cotton on a broach to get the shape and length of the root. Force the wire in as far as possible and twist it off. I had a case of abscess recently. Treated with carbolic acid and filled at once with lead, and the case was cured in two weeks.

Dr. Morrell: Nearly all the dentists in Louisville are using lead. It is too soft to draw through a draw-plate. It being so soft is favorable to its adaptation to inequalities. It can be forced where gold will not go.

Dr. Leslie demonstrated the use of crystal gold by filling a cavity.

The sixth subject was next considered: "Subjects connected with mechanical dentistry, including irregularities."

Dr. C. R. Taft: In correcting irregularities, I prefer to move the teeth gradually, more slowly than is the practice of some dentists. In expanding the arch I should expect to be three or four months in accomplishing the object. Dr. Keely accomplishes it in two weeks.

Dr. Taft exhibited models showing a case of irregularity corrected. The irregularity, which was very marked, had been successfully reduced. The girl was fourteen years old and was under treatment five months. She was able during that time to eat as usual and during that time had never complained of soreness. Some of the teeth had been moved a quarter of an inch. The appliance used was a rubber plate, into which pegs were inserted as required. No teeth were extracted. As the teeth moved the lower antagonists preserved a perfect occlusion, adapting themselves to the change.

Dr Van Antwerp: We have numerous instances in nature which illustrate the necessity for rest after severe effort, as for instance in parturition. The womb contracts forcibly for a time, and then there is a cessation, to allow the system to rally its forces for a new effort. We should take a hint from this and in moving teeth give them occasional rest. I never practice correcting irregularities by rapid stages.

Dr. Morrison: Expand the arch so as to make it accommodate all the teeth that nature gives. Avoid extracting if possible. There is sometimes much difficulty in expanding the the lower jaw to make it correspond with the upper.

Dr. Smith: I think the key-note was struck when the point was made that nature requires intervals of rest. There is often too much hurry.

Aug-3

Dr. McKellops stated a case of a girl of 12 years. The inferior anterior teeth shut outside of the superior incisors. She had been advised to have the lateral incisors extracted. He finally undertook the case and effected a correction of the irregularity without extracting a tooth.

Dr Keely demonstrated his method of procedure in a case which he adduced. A report would be unintelligible without the accompanying models.

He affirmed that the operation of regulating a set of teeth could be undertaken with a reasonable prospect of success at almost any age. Of course more patience and perseverance will be required in attempting it in the case of older persons. The younger the patient is, the more rapidly the dentist can operate. He has accomplished the object inside of twenty-eight days. A single tooth may sometimes be moved in from three to five days. He had seen the alveolus fractured in the case of a patient 26 years old by too rapid wedging.

Dr. Baxter: I would inquire how to correct a protrusion of the incisors, where there is a spreading and a projecting of the teeth. The upper teeth close inside of the lower or the reverse.

Dr. Goddard: I was called upon by a girl 16 years old, many years ago. Her upper teeth struck inside the lower, chin protruded, and her physiognomy reminded me of a bull dog. The space between her teeth when her mouth was closed was large enough to admit a finger. At that time I had not read anything on irregularities. Told the girl's father that I thought I could correct it, but that it would probably be a painful operation and a tedious one. I first extracted the second bicuspids of the lower jaw and made a gold plate to extend around the teeth and drew back the incisors until the spaces of the extracted teeth were closed up. The apparatus was constructed with a screw which could be tightened from time to time. After two or three months, the teeth became very sensitive indeed and were all loose. I hesitated. Then I made a plate and band and worked at the upper teeth for a while, drawing them out as far as I wanted them, securing a perfect articulation. Held the teeth in position a while and then removed the apparatus. They maintained their position The molars, which at first did not articulate at all, were now in perfect articulation.

Held the teeth in position for a while and then removed the apparatus. They maintained their position. The molars which at first did not articulate at all were now in perfect articulation.

That was forty years ago. I often see the woman. She is a different looking person, indeed is now quite comely. She says they are my teeth, not hers.

I was eleven months effecting the correction, and my fee was $25. (Laughter.)

I do not think there is any remedy for irregularity when the molars articulate perfectly and the anterior teeth are spread and are too short to be brought together.

Dr. Reid: Dr. Keely made a demonstration before the class in this lecture room recently, which illustrates that condition. The second molar below and a portion of the first molar above were the only ones in articulation. The first lower molars had been extracted some time before he undertook the case. He first wedged forward the lower molars somewhat and then extracted the second molars above.

He has advocated the extraction of the first molar in such cases. I told him I thought he made a mistake in this case.

Dr. Clayton: I have such a case as Dr. Baxter speaks of. The front teeth do not come together by a quarter of an inch. The lady has become very deaf. She has been under treatment for a long time without benefit. It is a question whether the deafness is not an effect of the abnormal condition of her teeth.

Dr. Dennis: I corrected an irregularity for a boy 12 years old. The upper anterior teeth protruded very considerably I drew them backward by means of a plate and ligatures. The mouth being constricted from side to side, I fitted the plate in pretty tight so as to spread the bicuspids.

Dr. Van Antwerp: I have seen two cases of blindness resulting from decayed teeth. It seems to me quite probable that if decay in a tooth could cause such trouble and exert such an influence over the optic nerve, that it might affect the auditory nerve as well, as there is immediate sympathy between the branches of the fifth pair of nerves and the organs of special sense.

Dr. Baxter: Disease of the antrum may be the cause of deafness, by causing inflammation of the Eustachian tube.

Dr. Morrison: I had a case in which the upper teeth closed within the arch of the lower. By expanding the upper jaw enough to throw the teeth over the lower ones, I found that the upper teeth were too short. They did not strike the lower by three-eighths of an inch at least. I arranged a skull cap and a chin piece with elastic bands to draw it up. The patient wore that three or four weeks. I think it was two months before I got it to operate to my satisfaction. Absorption set in under the teeth and the base was finally perfectly regulated.

Dr. Goddard: Why would not the natural articulation of the jaw answer the same purpose?

Dr. Morrison: It would if pressure were persistently kept up.

Dr. Osmond: *Undershot* jaws are a peculiarity of Welch people.

The subject of mechanical dentistry being under consideration, Dr. Baxter presented an impression cup of peculiar design made of wire and cloth.

Dr. Osmond: I have for years taken all my impressions in plaster. If the teeth are irregular and the cast breaks, I save the pieces and fit them together.

Dr. C. R. Taft reported favorably of the celluloid base. It is not to be compared with gold. Thinks it will yet be the best material for cheap work.

Dr Van Antwerp: I have been using celluloid ever since it was first introduced. Use gum teeth for permanent sets. Have had some trouble in cases where the arch of the mouth is deep. I obviate the difficulty in such cases by making a tin cast, beveled so as to enable me to take out one-half at a time. Work it just as I would rose pearl in such cases, using gum teeth. Do not think it advisable to use it as a continuous gum in all cases. Have had no cases returned to me. I dry out the flask thoroughly in a temperature of 215° to 220°

for half a day. As an attachment for a gold plate instead of a gold backing for the teeth, it does admirably. Never raise the temperature above 290° in working celluloid; a safety valve is not reliable; the thermometer should be attached to every celluloid apparatus.

Adjourned.

